# Green Micro- and Nanoemulsions for Managing Parasites, Vectors and Pests

**DOI:** 10.3390/nano9091285

**Published:** 2019-09-09

**Authors:** Lucia Pavoni, Roman Pavela, Marco Cespi, Giulia Bonacucina, Filippo Maggi, Valeria Zeni, Angelo Canale, Andrea Lucchi, Fabrizio Bruschi, Giovanni Benelli

**Affiliations:** 1 School of Pharmacy, University of Camerino, via Sant’Agostino, 62032 Camerino, Italy (L.P.) (M.C.) (G.B.) (F.M.); 2 Crop Research Institute, Drnovska 507, 161 06 Prague 6, Ruzyne, Czech Republic; 3 Department of Agriculture, Food and Environment, University of Pisa, via del Borghetto 80, 56124 Pisa, Italy (V.Z.) (A.C.) (A.L.); 4 Department of Translational Research, N.T.M.S., University of Pisa, 56124 Pisa, Italy

**Keywords:** agricultural pests, dengue, filariasis, insecticides, larvicides, mosquito control, stored product insects

## Abstract

The management of parasites, insect pests and vectors requests development of novel, effective and eco-friendly tools. The development of resistance towards many drugs and pesticides pushed scientists to look for novel bioactive compounds endowed with multiple modes of action, and with no risk to human health and environment. Several natural products are used as alternative/complementary approaches to manage parasites, insect pests and vectors due to their high efficacy and often limited non-target toxicity. Their encapsulation into nanosystems helps overcome some hurdles related to their physicochemical properties, for instance limited stability and handling, enhancing the overall efficacy. Among different nanosystems, micro- and nanoemulsions are easy-to-use systems in terms of preparation and industrial scale-up. Different reports support their efficacy against parasites of medical importance, including *Leishmania*, *Plasmodium* and *Trypanosoma* as well as agricultural and stored product insect pests and vectors of human diseases, such as *Aedes* and *Culex* mosquitoes. Overall, micro- and nanoemulsions are valid options for developing promising eco-friendly tools in pest and vector management, pending proper field validation. Future research on the improvement of technical aspects as well as chronic toxicity experiments on non-target species is needed.

## 1. Introduction

### 1.1. Micro- and Nanoemulsions 

Over the past decades, pharmaceutical, food and agricultural research has focused the attention on the development of delivery systems able to encapsulate, protect and deliver lots of different compounds. One of the most versatile tools is represented by colloidal dispersions, which are heterogeneous systems in which the inner phase is dispersed into a continuous medium. Micro- and nanoemulsions (MEs and NEs respectively) are self-emulsifying colloidal systems, having the internal phase usually smaller than 100 nm, dispersed in a liquid medium [1]. This characteristic enhances some physicochemical properties, i.e., stability and bioavailability. In fact, the small size of the internal phase allows the system to bypass the problems related to the gravity force, avoiding phenomena as creaming or sedimentation. Moreover, the low surface and interfacial tensions promote suitable spreading and penetration of the active compounds [2]. 

MEs and NEs are generally composed of an aqueous phase, an oily phase, a surfactant agent and a possible cosurfactant. For this reason, they are able to incorporate both hydrophilic and lipophilic compounds [3]. The choice of MEs and NEs components are strictly related to their application. For example, it is possible to select several oily phases between synthetic oils, (ethyl oleate, squalene and triglycerides), mineral oils and vegetable oil (e.g., olive, sunflower and soybean oil). Generally, the oily phase is used to solubilise and carry lipophilic molecules, but sometimes the oily fraction, as in the case of plant essential oils (EOs), can also be the active ingredient. EOs have been widely used in traditional medicine around the world since the Middle Ages, mainly for their antimicrobial and antioxidant properties.

A fundamental aspect about the formulation of EOs is the selection of suitable surfactant agents. The amphiphilic properties of a surfactant are represented by the hydrophilic–lipophilic balance (HLB) value. The choice of the suitable HLB value depends on the nature of the continuous phase. However, it should be desirable to select a surfactant with an intermediate value because it will partition between the aqueous and the oily phase, lowering the interfacial tension and conferring the optimal curvature of the layer, to guarantee the formation and stabilisation of the droplets. Depending on the chemical properties, surfactants can be divided into different classes: anionic, cationic, non-ionic and zwitterionic. The most diffused are polisorbates (anionic), such as Tween 80 (HLB 16.7) and Span 80 (HLB 8.6). In recent years there has been a growing interest in exploiting the surfactant properties of natural products such as polysaccharides, proteins (lectin) and sugar esters, which are desirables for the development of eco-friendly formulations. MEs and NEs have been deeply investigated, since they possess some practical advantages: easiness of formulation, industrial scale-up and high potential for use in several applications. 

**Figure 1:**
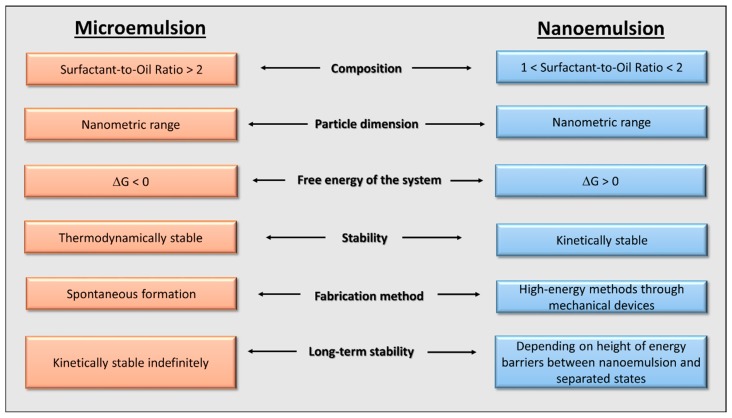
Comparison of the main physicochemical properties between micro- and nanoemulsion.

Apart from the terminology, these two systems present some substantial differences that it is necessary to highlight to better understand the mechanisms of their formation: (i) physicochemical behaviour, (ii) properties and (iii) applications. A summary of the main features of MEs and NEs is reported in Figure 1. 

First, it is important to highlight that, despite the prefixes ‘micro’ and ‘nano’ define two different orders of magnitude, i.e., 10^−6^ and 10^−9^, respectively, the size of the dispersed phase (generally oily droplets) for both of these two systems fall in the nanometric range. According to the literature it is not possible to exactly define a range of particle size distribution, since different authors report different results within the nanometric order of magnitude [3,4]. In any case, it has been reported that MEs are characterised by a smaller size of the dispersed phase respect to NEs [5,6]. 

ME has been defined as “a system of water, oil and amphiphile, which is a single optically isotropic and thermodynamically stable liquid solution” [7]. Introduced for the first time in 1944 by Hoar and Schulman, MEs were initially investigated for oil recovery from underground reservoirs [8,9]. Furthermore, the interest around them spread into several application fields. MEs were studied in detail in the pharmaceutical field as promising drug delivery systems for lipophilic compounds. As previously mentioned, they show several advantages such as solubilization of lipophilic compounds, enhancement of physicochemical stability respect to the related macro-systems (emulsions), improvement of the active ingredients bioavailability, achievement of a controlled drug delivery system, easiness of preparation and scale-up [10]. However, their real use is limited by the high amount of surfactant requested for the formation of such system, being these agents irritant against mucous membranes and potentially hazardous for the environment [11,12].

On the contrary, one of the most important advantages of NEs is the presence of low amounts of surfactant, generally less than 10%, compared to almost 15% in MEs, and a low surfactant-to-oil ratio (SOR) necessary for their formation, that is, >2 in MEs and comprised between 1 and 2 in NEs [2,5,6]. Briefly, NE is defined as “a thermodynamically unstable colloidal dispersion consisting of two immiscible liquids, with one of the liquids being dispersed as small spherical droplets (r < 100 nm) in the other liquid” [5]. It can be considered as a conventional emulsion, with the only difference of a smaller size of the dispersed phase. However, the most influential parameter varying in these two nanostructured colloidal dispersions is their free energy, conferring them different features in terms of preparation, formulation and stability. 

As reported in the previous definitions, MEs are thermodynamically stable while NEs are kinetically stable. This is due to the free energy possessed by the separate state (oil + water) respect to the colloidal systems. MEs are energetically favoured, with Δ*G* values lower than the respective separate phases. On the contrary, NEs (oily droplet in water) possess higher free energy than those of the separate phases, water and oil. 

The preparation methods of MEs and NEs are a direct consequence of this aspect. In fact, being the formation of MEs favourable, they can be obtained spontaneously by mixing oil, water and surfactant, without any external energy input. However, the application of magnetic stirring or heating could be convenient to expedite the process in order to overcome the kinetic barriers.

The energetic process that drives the MEs formation is based on the following formula [13].

Δ*G* = γ Δ*A* − *T* Δ*S* (1)

where Δ*G* is the free energy of the final system (ME), γ is the interfacial tension oil–water, Δ*A* is the variation of the interfacial area, T is the temperature and Δ*S* is the variation of the system entropy.

Briefly, Δ*G* must be negative so that a process occurs spontaneously. Since Δ*A* is very high in a ME (because of the formation of lots of small oily droplets that increases the interfacial area), this process is promoted by a very slow interfacial tension (γ) and by the entropy of the system that rises for the transition of the separate phases into only one containing a large number of particles; this allows obtaining a negative Δ*G* value.

The formation of MEs is strictly dependent on the sensitive SOR and, to determine the optimal one, is used to build a pseudoternary phase diagram. This kind of system, in fact, needs a very low interfacial tension and a favourable packaging of surfactant molecules, given by the relative interaction between their hydrophobic tails and the oil phase. This allows the formation of a fluid film at the oil–water interface [14]. Usually, the addition of cosurfactant agents is required, generally alcohols, to facilitate this phenomenon, useful to reduce the amount of surfactant as well [15]. Being MEs dynamic systems, we have to take into account that the interface is continuously subjected to a rearrangement of its structure and to the Brownian motion of the internal phase, with a possible variation of its radius [16]. 

Since NEs are thermodynamically unstable, the free energy of the systems, Δ*G* (Formula (1)), will be always positive. Thus, to exceed this value, an external energy input results to be necessary. 

Depending on the physicochemical mechanisms, the methods used for NE preparation can be divided into high-energy and low-energy methods. The first ones use mechanical devices able to provide the force needed for the disruption of the dispersed phase into very small droplets, in the range of nanometres (r < 200 nm). Generally, NE formation follows a two steps procedure. In the first phase there is the formation of a macroemulsion through a mechanic stirrer. In the second one the macroemulsion is converted into a NE.

The most common devices used for this process are microfluidizer, sonicator, and high-pressure homogenizer. This last device uses high pressure value to pump the macroemulsion in a very narrow orifice that promotes the breaking of big droplets into smallest ones. The same result is achieved through ultrasound waves that lead to the dispersion process by means of cavitation phenomenon.

Although these approaches seem to be robust, they show some limitations concerning costs, process implementation and industrial scale-up [17].

On the contrary, low-energy methods are simpler, cheaper and more effective in producing smaller droplets. However, they require an accurate knowledge of the process parameters, showing some limitations in the ingredients and conditions [6,18].

Generally, low-energy methods are based on the phase inversion, transforming a W/O macroemulsion into an O/W NE through the variation in composition (emulsion inversion point (EIP)) or temperature (phase inversion temperature (PIT)). At the inversion point, the interfacial tension is so low that very fine droplets can be obtained, only with the support of low energy input.

Briefly, the phase inversion due to the PIT method is linked to the presence of surfactants that, based on a temperature change, modify their affinity for the hydrophilic or lipophilic phase. With EIP method there is a modification in the composition (water, surfactants, electrolytes) of the final system, which leads to a variation of the lipophilic-hydrophilic balance, with a consequent change in the curvature of surfactant layer. The free energy of the system influences the long-term stability behaviour as well. MEs should remain stable indefinitely, if the initial conditions about the chemical composition and storage will keep unchanged.

NEs, instead, will remain in a metastable state that will guarantee the stability of the systems if the energy barrier between the two different energy states remains high enough to avoid the reversion of the system and the phase separation. This occurs because of such instability phenomena such as coalescence, flocculation and Ostwald ripening, which, bringing growth of droplets, lead to creaming. It represents the migration of the dispersed phase influenced by buoyancy.

Coalescence is due to the merger of small droplets into bigger ones, while, in flocculation, droplets become very closer to move as a unique phase. These phenomena are related to the surfactant layer on the droplets surface that guarantees the steric stabilisation as much as the thickness of the layer is comparable with the droplets size. For this reason, NEs are not particularly affected by coalescence and flocculation, as compared to a traditional emulsion.

On the contrary, NEs are more prone to Ostwald ripening. This phenomenon can be defined as: “the process of disappearance of small particles or droplets by dissolution and deposition on the larger particles or droplets. The driving force for Ostwald ripening is the difference in solubility between the small and the large particles. The smaller particles (with higher radius of curvature) are more soluble than the larger ones (with lower radius of curvature). With time, the smaller particles or droplets dissolve, and their molecules diffuse in the bulk and deposit on the larger ones. This results in a shift of the particle or droplet size distribution to larger values” [19]. It is a thermodynamic process, being larger particles energetically favoured over the smaller ones.

Since the aqueous solubility of the oily droplets strongly influences the occurrence of this phenomenon, a suitable solution could be the addition of non-polar compounds that condition positively the distribution of the droplets in the oily phase. Some of the most used “ripening inhibitors” are medium-chain triglycerides (MCT), corn oil and sunflower oil [20,21,22].

Concluding, some of the most influential parameters on the NE stability are:

(i) The SOR and relative concentrations; they influence the interfacial tension. It is not possible to stabilize a fixed relationship between these parameters because they are strictly related to the nature of the compounds that confer unique properties to the systems, which, in turn, differ from each other.
(ii) The ionic strength of the dispersion medium; it affects the repulsive forces between the droplets of the dispersed phase. As the ionic strength increases, the repulsive forces decrease and the systems will be prone to instability.
(iii) The solubility of the dispersed phase; it allows droplets to move towards the continuous phase with the appearance of Ostwald ripening.
(iv) The temperature; it affects the solubility with the above-mentioned consequences. Moreover, it influences the energy balance of the system as well.

### 1.2. Applications

Thanks to the previously mentioned advantages, such nanosystems have been widely exploited in different fields as a tool for oil recovery, fuel and reaction medium in chemical applications [23,24,25]. However, in this section we are going to focus the attention on their applications in food, agrochemical, cosmetic and pharmaceutical fields.

About the food area, they have been developed to improve and extend the use of low water-soluble compounds or food-derived bioactive compounds with poor bioavailability. Such delivery nanosystems seem to be a suitable tool to solve this kind of problems. A significant example has been reported by Yu and Huang [26]. They demonstrated that curcumin showed a 9-fold increase in oral bioavailability when encapsulated into NEs. Moreover, it was faster digested as well, through lipolysis, respect to the unformulated compound. In the last years, NEs have been considered as a fundamental tool for the delivery of functional substances in functional foods or fortified beverages such as fatty acids, polyphenols, vitamins, micronutrients, antioxidants and others [27]. For example, O/W NE was exploited in order to encapsulate and deliver Omega-3 fatty acids in yoghurts [28].

Being extremely stable in a wide range of pH, MEs and NEs are very useful for encapsulating nutrients and protecting them from environmental conditions such as temperature or light-mediated oxidation and from possible transformation by means of enzymatic reactions and hydrolysis [29]. They formulated a valid solution to maintain suitable organoleptic properties of foods and beverages. In fact, MEs and NEs can encapsulate volatile molecules and control the release of flavours. Moreover, they can be used to prevent contamination of products and to prolong their shelf-life, both directly, for example by adding a preservative NE inside food, or indirectly, by functionalizing the packaging system in the same way [30,31]. Besides these advantages, MEs and NEs in food chain show some limitations, due to the nature of the components. For examples, in a food product the oily phase should be a triglyceride. Since the solubilization of a long chain triglyceride (LCT) is hard to obtain, it should be preferable to choose between a medium and short chain triglyceride [32].

Actually, the real limiting step in food grade nanosystem formulation is related to surfactant, because many of them are not allowed for human consumption or just at very low concentrations. Some of the admitted ones are sugar esters, monoglycerides, lecithins, glycolipids, fatty alcohols and fatty acids [33]. This issue is, nowadays, a great object of study. A large number of authors in fact, through the building of pseudoternary phase diagram of food grade components, tried to find suitable and stable formulations based only on food-grade compounds [32].

Regarding the pharmaceutical field, modern technology is progressing toward developing efficient drug delivery tools, with particular attention to an increase of bioavailability, a controlled release of the drug, a targeted biological effect and good storage stability over time. All these goals could be pursued by the exploitation of MEs and NEs. Being composed of hydrophilic and lipophilic domains, they are versatile systems able to incorporate and solubilise drugs of both natures. Araya et al. proved that MEs enhanced the oral bioavailability of poor water soluble drugs, as Ibuprofen and Ketoprofen, increasing their solubility and their plasma concentration from 60 to 20,000 times [34].

Since MEs/NEs can raise the bioavailability, the administered dose of drugs could be reduced minimizing possible side effects. These formulations behave as controlled release tools, both in O/W and W/O systems. In fact, in the first case, the oily phase acts as reservoir of active compounds, while when the oil is the external phase limits the diffusion [35]. However, the rate of drug release is influenced by the composition of the environment, such as pH and ionic strength, and features of the nanosystems, i.e., droplet dimension, type of MEs or NEs, nature of the drug and route of administration. Moreover, a limiting step is represented by the ability of the drug to cross the biological barrier, such as mucosa cells or skin [36].

Oral delivery of such nanosystems should be very useful to carry on poor water-soluble drugs, since they allow to overcome the dissolution issue on gastric fluids, which generally is strictly related to bioavailability. Moreover, they reduce the hepatic first-pass metabolism favouring the passage of the drugs in the bloodstream [35]. The small size of the internal phase and the presence of surfactants improve the drug absorption in the gastrointestinal (GI) tract, in the first case, enhancing the permeability of biological barriers, and, in the second case, promoting a wide and deep distribution [3,37]. 

Yin et al. showed how a ME, composed by Capryol 90 (oil), Cremophor EL (surfactant) and Transcutol (cosurfactant), increased the bioavailability of docetaxel, as compared to the related commercial product, after oral administration in rats. This result was obtained through the cumulative effect of enhanced drug solubility, improved permeability and inhibition of P-glycoprotein (P-gp) efflux [38]. Thanks to their low viscosity and possibility to be sterilised by filtration, MEs and NEs are very favourable in parenteral administration as well [4]. Moreover, they showed an appreciable physical stability in plasma [39]. 

Both O/W and W/O systems are suitable for parenteral formulations. Generally, O/W systems are used to deliver lipophilic compounds in order to obtain a controlled release of the drugs. Thus, they are administered by the intravenous, intramuscular or subcutaneous routes. On the contrary, W/O systems, applied as subcutaneous or intramuscular administration, are suitable to encapsulate hydrophilic drugs in order to obtain prolonged release delivery systems [4]. 

Dordevic et al. optimised a risperidone-based NE and monitored the pharmacokinetic parameters of the active ingredient. After intraperitoneal administration in rats, they obtained a 1.2–1.5-fold increase of bioavailability, 1.1–1.8-fold decrease in liver distribution, and 1.3-fold increase of brain uptake of risperidone as compared to the drug solution [40]. MEs and NEs are widely studied and used for topical, ocular and nasal administration as well [13,41]. The topical route has been investigated mainly in the cosmetic field, exploiting these systems in order to obtain a better penetration of the active molecules through the skin barrier [42]. 

Intranasal route should be exploited to deliver active molecules directly on the brain. Vyas et al. developed a mucoadhesive clonazepam-based ME for the epilepsy treatment. The concentration of this molecule in the brain was found to be 2-fold higher when compared with intravenous administration, indicating an enhanced distribution and bioavailability of the active ingredient in the site of action [43]. 

As in other nanosystems, functionalisation of MEs and NEs allows to build up targeted drug delivery systems, which are able to address the activity mainly in a desired target site.

Shiokawa et al. reported the formulation of aclainomycin A, a lipophilic antitumour-antibiotic drug, through a ME linked to folate molecules. They showed that, the use of folate, helpfully modified with PEG molecules, can be considered as an effective strategy to target MEs on tumour cells [44].

**Figure 2:**
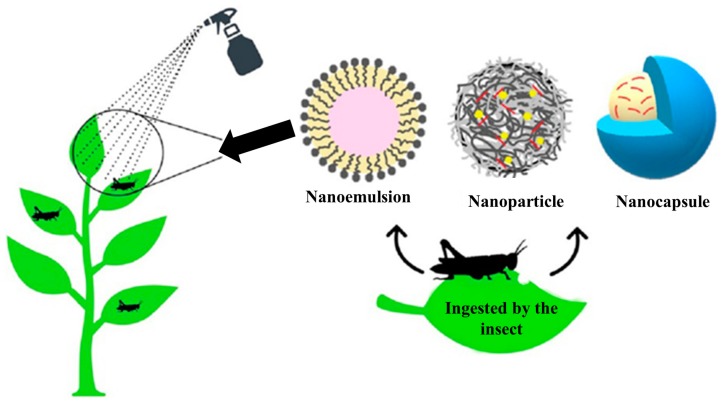
The most used nanosystems in insect pest control (adapted from Medina-Pérez et al. [45], with permission of Elsevier, 2019).

Another interesting field of application of nanosystems is the agricultural one. In particular, nanotechnology is starting to revolutionise the pest management, providing innovative tools, i.e., nanoemulsions, nanoparticles and nanocapsules for the delivery of pesticide compounds (Figure 2). 

Among several nanodelivery systems, MEs and NEs are the easiest ones to handling and formulate. In particular, they are necessary in the presence of compounds with low water solubility that require a delivery system for their application in the field [46]. Du et al. carried out a systematic study about the formation of O/W NE based on methyl laurate as oil phase and alkyl polyglycoside and polyoxyethylene 3-lauryl ether as surfactants [47]. Moreover, they evaluated the effect of β-cypermethrin on the stability and physicochemical properties of the system.

The encapsulation process improves the physicochemical stability of pesticides and prevents the degradation of active agents [48]. Song et al. (2009) proved that the encapsulation of triazophos—an organophosphorus insecticide—is able to prevent the hydrolysis of the active compound [49]. In terms of bioactivity, these compounds result to be more effective. Nanosystems are able to ensuring their release to the target site, also providing a controlled release of the molecules at the site of action and thus reducing the required concentration of applied pesticides [2,49]. Moreover, thanks to the small size of the dispersed phase, the active compounds could improve their spreading, deposition and permeation on the target site.

## 2. Green Micro- and Nanoemulsions

In the last years, the growing interest of the global community on the planet fate is leading towards a more responsible and sustainable exploitation of natural resources. In particular, the worth of plants, as primary sources of ingredients for the realisation of a great variety of products, has been revaluated. In fact, some plant-based materials offer superior performance characteristics as compared to the synthetic ones. Nowadays, they have started to be applied in several fields such as pharmaceuticals, nutraceuticals, cosmetics and agrochemicals. Relying also on longstanding uses in the traditional medicine systems, they are generally employed as essential oils (EOs) and extracts, acting as flavouring agents, dyes, fortifying agents in functional foods or actual active ingredients [50]. 

EOs are mixtures of volatile and lipophilic molecules (mainly terpenoids and phenylpropanoids), produced in secretory structures of aromatic plants, in particular those belonging to angiosperms, such as Apiaceae, Asteraceae, Geraniaceae, Lamiaceae, Lauraceae, Myrtaceae and Verbenaceae, as products of their secondary metabolism [51].

EOs have been widely employed in the flavour and fragrance industry. They also find industrial application in foodstuffs (e.g., soft drinks, food and packaging) and cosmetics (e.g., perfumes, skin and hair care products). Regarding their medical properties, EOs are mainly used as antimicrobial agents.

Recent studies have attested pesticide properties of several EOs, natural pure compounds and extracts. The use of plant sources in crop protection dates back to 2000 years ago [52]. However, in the 20th century a wide spread of synthetic pesticides started to take hold. They were favourable thanks to a high and long-lasting efficacy. If, on the one hand, they increased crop yield, on the other hand, their overuse led to toxic effects on humans and the environment with occurrence of resistance in pests [53,54,55].

The current limitations of their use are pushing discovery and development of less harmful products. One of the most promising solutions is the exploitation of plant-based pesticides. In fact, if the synthetic pesticide market is expected to decline by 1.5% per year, biopesticides have been estimated to reach the 20% of the pesticide market by 2025 [56,57].

The oldest and most widely used biopesticide is pyrethrum, a pure compound derived from the dried flowers of *Tanacetum cinerariifolium* (Trevir.) Sch.Bip. (Asteraceae) [58]. Actually, it has taken around 80% of the biopesticide market [59]. By virtue of its low toxicity against both mammals and environment, it presents a high safety profile [60]. However, its synthetic derivatives, also known as pyrethroids, have been designed to emulate the activity of the natural molecule. Despite their efficacy, they showed to be hazardous for the environment because of their long-lasting effects and high toxicity against non-target organisms [61]. 

Nicotine and the other alkaloids of tobacco represent another class of botanical pesticides. They act on the nervous system of pest, mimicking the neurotransmitter acetylcholine. Their use is now declined for their proved toxicity on human beings. The same problem has been observed for rotenone, isolated from *Derris elliptica* (Wall.) Benth. roots. Even though it is one of the most effective biopesticides, its high toxicity towards aquatic organisms and mammals deeply limited its use [62]. 

Neem (*Azadirachta indica* A. Juss.) is source of a very interesting compound, azadirachtin, a limonoid with considerable pesticide activity. It has shown bactericidal, fungicidal, and insecticidal properties, acting as a feeding and oviposition deterrent and as a growth inhibitor [63]. A fundamental aspect is its safety profile: no persistence in soil, no adverse effects on water or groundwater organisms, no toxicity to mammals [64,65].

Eco-friendly alternatives in biopesticides include the wide group of EOs. One of the most promising aspects in the exploitation of EOs is their lack of toxicity on mammals; they are generally harmless for the environment when compared with synthetic pesticides [66]. Their safety profile is guaranteed by the fact that most of EOs have been recognised as Generally Recognised As Safe (GRAS) substances by the Food and Drug Administration (FDA) and by the Environmental Protection Agency (EPA) of the United States [67]. For these reasons, a possible residue of EO-based pesticides on crop does not constitute a risk for human health.

It has been reported that EOs, such as thymol-containing EOs or EOs compounds, such as eugenol or α-terpineol, showed LC_90_ values two or three order of magnitude higher as compared to synthetic commercial products, such as endosulfan, against Juvenile Rainbow Trouts [68]. Pavela et al. reported that Apiaceae EOs have no toxicity against non-target organisms, as adult microcrustaceans *D. magna* and adult earthworms *E. fetida*, unlike α-cypermethrin that, even in much lower concentrations, caused almost 100% mortality [69].

Beyond the proofs about their safety, in the last years several studies have been carried out on the pesticide efficacy of EOs. Results showed that such substances exert a marked activity against pests, both in direct and indirect way. They act as chemosterilant, fumigant, ovicidal and repellent agents, altering growth, development and feeding behaviour [70,71,72,73]. In a recent review, Pavela collected the results published about the pesticide activity of EOs deriving from around 122 different species. Their efficacy could be expressed by an exciting data: 77 EOs showed LC_50_ < 50 ppm [74]. Their bioactivity is strictly linked to the presence of different compounds present in the mixture of each EO, monoterpene and sesquiterpene hydrocarbons, phenolic monoterpenes, oxygen containing mono- and sesquiterpenes and phenylpropanoids [75].

The main mechanism of action is linked to the ability of EOs to interfere with the cell membrane. Their accumulation leads to the disruption of the cell wall, leakage of the cellular contents and perturbation of homeostasis [76,77]. All these alterations lead to cell death. It has been reported that several EO constituents act in this manner [78,79]. Nevertheless, EOs, as well as plant extracts, are able to interfere with the nervous system of pests and vectors, inducing even death [80].

For example plant extracts, in particular alkaloids, can act at different levels of the pest nervous system [81]. They can function as competitive inhibitors of the acetylcholinesterase (AChE) enzyme, with consequent accumulation of the neurotransmitter in the synapses, followed by a state of permanent stimulation of the postsynaptic membrane [82]. Moreover they could be antagonist of GABA receptors as well, causing hyperexcitation, convulsion and death of the pest due to reduction of neuronal inhibition [83]. However, the most important target site of EOs is the octopaminergic system [80,84]. Octopamine is a neuromodulator and the absence of octopamine receptors in mammals is the factor that determines the distinction between target and non-target organisms. Acting on the octopaminergic system, the active compounds will be harmless for non-target organisms [72,85]. 

In addition to the above-mentioned advantages on the exploitation of EOs as biopesticides, a fundamental aspect is their synergistic effect. Synergism occurs in EOs since they are a mixture of 20–60 compounds, where all the components cooperate to enhance the bioactivity [86,87]. This results in a high efficacy since they act with different and complementary mechanisms of action and the combined effect is usually higher than those of the single components, allowing the reduction of the effective dose. Moreover, the mutual synergism represents a suitable tool to fight the development of resistance phenomenon, which is common with synthetic pesticides, that normally have only a target site [75].

Since EOs showed to be among the best candidates as botanical pesticides, we can ask why its commercial spread is still limited. The reason is strictly linked to their physicochemical properties, such as lipophilic nature and thus poor water solubility, scarce stability, high volatility, thermal decomposition and oxidative degradation [88]. These aspects translate into reduced efficacy and handling difficulties [72,85]. Moreover, being volatile compounds, EOs show low persistence in the environment and a scarce accumulation in soil and water [89].

All these reasons are encouraging researchers to find out suitable solutions to protect and deliver EOs. Currently, the selected strategy is the encapsulation method. Encapsulation is a process through which an active compound is coated or entrapped into a matrix. In this way, the bioactive molecule is isolated and protected by the matrix from the surrounding environment and its release depends on the external conditions and the matrix nature as well [88].

In this respect, in the last years nanotechnology revealed to be the best approach for the exploitation of EOs, allowing to overcome the limitations related to their use [48,90,91,92,93,94,95]. Although nanotechnology represents an innovative tool able to revolutionise pest management science, it remains a big, but exciting, challenge. An example of EOs stabilisation has been reported by Cespi et al. [96]. They found a suitable solution allowing the use of *Smyrnium olusatrum* L. EO, an oil difficult to handle for stability problems related to the high concentrations of its main constituent, isofuranodiene, which easily undergoes crystallisation. After a systematic study based on an experimental design, they found the best ME capable of encapsulating and protecting EO thanks to the presence of ethyl oleate that avoids the crystallisation issue. Moreover, this formulation proved to be stable over one year and maintained unchanged the bioactivity of EO. Pavela et al. used the same strategy to vehiculate isofuranodiene, the main active compound of *S. olusatrum* EOs [97]. Isofuranodiene-based ME (0.75%) has been tested against *Culex quinquefasciatus* Say showing potent larvicidal effects, with LC_50_ value of 17.7 mL·L^−1^.

The advantage of MEs and NEs to deliver EOs is not only related to the enhancement of the physicochemical stability but also to the improvement of bioavailability [2,49]. For this reason, the bioactivity of EO-based nanosystems is often higher than those of free EOs. Osman Mohamed Ali et al. carried out a study on the encapsulation of neem and citronella EOs in O/W NEs, to exploit their pest control properties. Stunning *in vivo* results were obtained towards phytopathogenic fungi *Rhizoctonia solani* (Cooke) Wint. and *Athelia rolfsii* (Curzi) C.C. Tu & Kimbr.; EO-based NEs showed exceptional effectiveness, which was higher than those of free EOs [98]. The higher activity of EO-based MEs compared to free EOs (*Trachyspermum ammi* (L.) Sprague ex Turrill, *Pimpinella anisum* L. and *Crithmum maritimum* L.) has been also demonstrated by Pavoni et al. on different species of bacteria and fungi [99]. Moreover, Liang et al. tested the antibacterial activity of peppermint EO NE and the relative free EO on *Listeria monocytogenes* and *Staphyloccoccus aureus* [100]. Although they showed comparable MIC values*,* the surprising difference was related to the long-term inhibition growth given by NE. Such formulation, by increasing the stability and solubility of EO, was capable of establishing a sustained release. The dispersed phase in fact acts as a nanotank releasing active ingredient over time [21].

Furthermore, the small size of the internal phase improves mobility and penetration with an increase of the activity, and the high surface area of the oily drops enhances the efficacy [101]. Salvia-Trujillo et al. demonstrated the advantageous bioactivity of EO-based nanosystem as compared to the related coarse emulsion [102]. In this case, the difference has been made by the size of the oily droplets, highlighting once again the great advantages generated by such nanosystems.

More explicative examples of EO-based MEs/NEs as biopesticides will be reported in-depth in the following sections.

## 3. Green Micro- and Nanoemulsions as Insecticides

### 3.1. Hemiptera

Hemiptera is an order of insects comprising ~68,000 species. Some of them, including many aphids, are important agricultural pests, damaging crops by the direct action of sucking sap, but also harming them indirectly by being the vectors of bacteria, phytoplasmas, spiroplasmas and viruses. They often produce copious amounts of honeydew which encourages the growth of sooty mould. Significant pests include the cottony cushion scale, a pest of citrus fruit trees, the green peach aphid and other aphids which attack crops worldwide and transmit plant diseases. Although several studies have been reported on the activity of EOs against Hemiptera species, only few authors investigated their effectiveness on the same target when encapsulated into MEs or NEs [103,104,105,106].

Among the few examples available, Fernandes et al. developed an insecticidal NE based on *Manilkara subsericea* (Mart.) Dubard extract [107]. The efficacy of hexane-soluble fraction from ethanolic extract of *M. subsericea* on *Dysdercus peruvianus* has been previously reported by the same authors [108]. *D. peruvianus* is an Hemiptera species (Pyrrhocoridae) that acts on cotton crops causing huge harvest losses [109]. Since the apolar fraction of the extract is water insoluble, the exploitation of NE technology seemed to be a favourable strategy. After a wide screening on the suitable HLB value of surfactants and the mean droplet size, the following NE composition has been chosen: 5% of *M. subsericea* extract solubilised in 5% octyldodecyl myristate (oil phase) and 5% of surfactants (sorbitan monooleate/polysorbate 80). This NE, characterised by mean droplet size of 155 nm and PDI value of 0.15, proved to be a good insecticide. In fact, it showed its activity since the first day of treatment (12% of mortality), that was sustained over time, with a mortality index of 66% of the insect population after 30 days. Moreover, the safety of this NE was confirmed noting the lack of effects against acetylcholinesterase as well as no acute toxicity on mice.

As said before, aphids represent ones of the world’s major insect pests, causing serious economic damage to a range of temperate and tropical crops. This ranges from grain crops and brassicas to potato, cotton, vegetable and fruit crops. For this reason, the investigation on botanical remedies to manage these pests gained great importance and generated several studies on a wide number of EOs and aphids species [110]. Santana et al. (2012) tested the activity of *Thymus vulgaris* L. and *Lavandula latifolia* Medik. on different aphid species, namely *Rhopalosiphum padi* (L.) and *Myzus persicae* Sulzer [111]. Isman (2000) evaluated the fumigant toxicity of four EOs on *Aphis gossypii* Glover, the pest that affects mainly cotton crops, as well as a variety of plants such as citrus, coffee, cocoa, pepper, potato and many ornamental plants [85,112]. On the same target Kalaitzaki et al. tested a formulation of natural pyrethrins, a combination of six esters extracted from the flowers of *T. cinerariifolium* [113]. They solubilised pyrethrins in lemon oil obtaining, initially a W/O ME that was suddenly diluted in water, leading to the formation of an O/W NE. Results about insecticidal activity showed lower LC_50_ and LC_90_ values of pyrethrin-based NE as compared to those of pyrethrum commercial products (761.8 vs. 965.5 mg/mL and 4011.2 vs. 5224.0 mg/mL, respectively).

Pascual-Villalobos et al. performed a wide screening of the repellence activity of 10 EOs and 18 pure compounds against *R. padi*, the major pest of cereal crops on a world scale [114,115]. To face the volatility issue related to the nature of EOs, authors encapsulated the most active ones in NEs, in particular aniseed and peppermint EOs, as well as geraniol, *cis*-jasmone and farnesol. The effectiveness of NEs were evaluated in terms of repellence (RD_50_ and RD_90_) and mortality after 24 h. Interestingly, some results showed that the smaller were the oil droplets the higher was the repellence activity. In particular, citral-based NE at 2%, having a particle size of 99 nm, showed a repellence index of 66, while the same formulation with larger particles (816 nm) exerted low activity.

### 3.2. Mosquitoes

Mosquitoes are the vectors of pathogens and parasites of medical and veterinary importance leading to the spread of diseases such as malaria, filariasis, dengue, yellow fever, Japanese encephalitis and Zika virus, just to cite the most important, some of them are lethal, especially in developing countries [116]. Thus, the effective management of these vector populations is a worthy challenge. At the moment, the main approaches to control their spread are: (1) killing adult species through the use of insecticides, (2) reduction of adults population interfering with their fecundity and oviposition or (3) killing mosquito young instars [74].

Although several pesticide products are available on the market, their dangerous effects on the environment along with the development of resistance bring to the need of new sustainable and eco-friendly tools. In the last years, research focused the attention on those EOs suitable as active ingredients in botanical larvicides. Pavela reported, from the literature, the activity of 122 EOs as mosquito larvicides [74]. Interestingly, 77 of them showed LC_50_ value < 50 ppm. Moreover, Pavela assessed the acute toxicity of 30 aromatic compounds of EOs against *C. quinquefasciatus* [87], which is the main vector of the lymphatic filariasis and has been investigated as a vector of Zika virus as well [117,118]. For this reason, several authors investigated the effect of different EOs encapsulated into MEs/NEs against this target. 

Oliveira et al. improved the water solubility of *Pterodon emarginatus* Vogel oleoresin through its dispersion in a polisorbate 80/sorbitan monooleate NE, at 1:1 oil–surfactant ratio [119]. This formulation caused the death of around 100% of *C. quinquefasciatus* larvae after an exposure time of 48 h at the concentrations of 100 and 200 mg/L, probably due to morphological alterations on the final abdomen segment of the larvae. Since the *P. emarginatus*-based NE did not exert any toxicity on the green algae *Chlorella vulgaris* Beijerinck, it can be considered an eco-friendly botanical product. The effect of EOs formulations on non-target organisms have been investigated in depth by Pavela et al. on the microcrustacean *Daphnia magna* Straus, the aquatic worm *Tubifex tubifex* (Müller) as well as the earthworm *Eisenia fetida* (Savigny) [69,97]. Moreover, they proved the larvicidal activity of MEs based on Apiaceae EOs, as those of *T. ammi*, *C. maritimum* and *P. anisum*, and on isofuranodiene, the major volatile compound of *S. olusatrum* EO, evaluating the chronic and acute toxicity on *C. quinquefasciatus*. These formulations showed remarkable efficacy, with LC_50_ values of 1.57, 2.23, 4.01 and 17.7 mL/L, respectively. 

Several studies have been conducted on the effectiveness of OEs-based MEs and NEs against *Aedes aegypti* L. larvae, the major vector of dengue and yellow fever. In particular, *Rosmarinus officinalis* L. and *Ocimum basilicum* L.-based NEs showed evident efficacy on larval mortality, in a time and dose-dependent manner [120,121]. Interestingly, several authors reported how the exploitation of nanotechnology in pest management could be useful to enhance, not only the stability of EOs, but also their efficacy as pesticide agents.

Balasubramani et al. [122] reported a study based on the larvicidal activity of *Vitex negundo* L. EO on *A. aegypti*. The encapsulated EO showed higher toxicity as compared to the free one, with lower LC_50_ and LC_90_ values. MEs and NEs, in fact, providing a higher dispersion of the lipophilic phase into an aqueous one, could increase the concentration of active ingredients dispersed at the interface leading to direct improvement of the interaction with the target [123].

An important parameter related to the EOs activity is the size of the oily droplets. In fact, Anjali et al. [124] observed that the smaller was the droplets size, the higher was the formulation efficacy. In particular, neem oil NE with a medium diameter of 31 nm caused the mortality of 86% of *C. quinquefasciatus* larvae after 24 h, while NEs of 93 and 251 nm showed a percentage of mortality of 73% and 48%, respectively. 

Sugumar et al. [125] compared the activity of *Eucalyptus globulus* Labill. EO encapsulated both in NE and bulk emulsion against *C. quinquefasciatus*. It was observed that, at the concentration of 250 ppm, NE caused 100% of mortality after only 4 h, while the bulk emulsion obtained the same result after 24 h. It is possible to suppose that the size reduction of oil droplets, and thus the increment of the surface area, lead to a better interaction and penetration of the active ingredients into the target organisms [126]. 

### 3.3. Stored Product Beetles

Cereal crops can be still considered a main food source for mankind [127]. However, their yield could be compromised by pest infestations during storage. This leads to an extensive loss of crops in term of quality and quantities. In fact pests, not only reduce the amount of grains, but also create suitable environmental conditions for the growth of moulds [128]. The most widespread insect of stored products is *Tribolium castaneum* Herbst, also known as the red flour beetle, which is able to release carcinogenic substances [129]. 

Botanical research found out several EOs able to fight stored product pests, in particular *T. castaneum*, acting through contact, fumigant, growth inhibitory, antifeedant and repellent actions [130]. Starting from this knowledge, several authors worked on the development of suitable formulation of EOs for their real application. Hashem et al. encapsulated *P. anisum* EO, known to be effective against *T. castaneum*, into a NE, in order to enhance its physicochemical properties [131]. 10% EO-based NE showed a mortality index of 81.33% after 12 days of exposure. Moreover, such system was able to significantly affect the development of progeny and reduce the grain weight loss (%). Morphological and histological evaluations showed that the EO-based NE adhered to several body parts and penetrated through the cuticle, causing cellular necrosis. On the same target, other authors tested EOs obtained from three species of *Achillea*, *A. biebersteinii* Afan., *A. santolina* Falk and *A. millefolium* E.Mey. [130]. They showed how the EO bioactivity depends on the kind of exposure and thus, the mechanism of action. In fact, fumigant toxicity proved to be more effective respect to the topical and contact ones. In particular, the EO-based NE showed significant higher fumigant toxicity as compared to the free EOs, with almost one order of magnitude lower LD_95_ values. Moreover, authors proved that these nanosystems were more effective, in terms of mortality, on adults as compared to larvae, although they strongly affected their growth and development.

Interestingly, Pant et al. added a new ingredient to EO-based NEs that was proven to enhance the effectiveness of the system [132]. They formulated 10% eucalyptus EO NE to test against *T. castaneum*, using karanja and jatropha aqueous filtrates (at increasing concentration from 20% to 60%) in place of water. Such filtrates, obtained from the de-oiled seed cakes, showed to possess insecticidal properties [133,134]. This study reported how the presence of aqueous filtrates improves the physicochemical properties of the formulations, reducing the medium size of the dispersed phase and the PDI value. Moreover, they enhanced the shelf-life of EO for long periods of time reducing its volatility. In fact, after two months, in presence of filtrates, the concentration of EO active ingredients remained unchanged, while in presence of water it decreased to 5%. 

*Eucalyptus globulus*-based NE has been investigated against the species *Sitophilus granarius* L., as well [135]. This formulation showed higher efficacy on this pest when compared with free EO. In addition, such NE showed to be safe, since it did not show mortality and did not cause biochemical alterations in rats. 

Choupanian et al. investigated the activity of neem oil NEs against *T. castaneum* and *Sitophilus oryzae* L., also known as the rice weevil [136]. Authors underlined as the effectiveness of a system could depend, not only on the presence and amount of active ingredients, but rather on the formulation parameters. In this case, the choice of the surfactant was carefully evaluated. In fact, polysorbate and alkylpolyglucoside have been compared. NEs obtained with polysorbate showed smaller droplets size and enhanced stability as compared to those containing the other surfactant. Moreover, by their reduced size, they showed higher activity since the active ingredient could penetrate the insect cuticle and come in contact with the target. Moreover, the study reported higher pest mortality of NEs as compared to commercial products and the crude oil extract. These results could be ascribed again to the reduced droplets size of the NEs that caused 100% of mortality in both species after 48 h. Although the previous mentioned species are the most common pests that affect stored products, researchers investigated EO-based NEs against other species as well, obtaining encouraging results about the effectiveness of such nanosystems on the preservation of cereal crops from the infestation of several different pests species [130].

## 4. Green Micro- and Nanoemulsions as Insect and Tick Repellents 

As detailed in the paragraph above, hematophagous insects act as main vectors of several diseases, such as Zika virus, dengue, malaria and yellow fever, causing more than one million deaths per year [137,138]. There is need of new specific drugs or vaccines to treat or prevent such diseases; however, one possible approach to control them is represented by reliable vector control tools, with proven epidemiological impact. One of the simpler ways to deal with this is the employ of repellent products. Repellents are chemical molecules able to prevent the arthropod landing on the skin and the consequent bite [139]. They act through a topical action forming a vapour layer having an intolerable odour for a given arthropod species, preventing its contact with human skin. It is desirable that such molecules do not penetrate in the bloodstream but, rather remain in the stratum corneum [140].

The ideal arthropod repellent should possess some key features: (i) broad spectrum of activity, (ii) long-lasting effect (>8 h), (iii) no toxicity for human being and environment, no skin irritation and low penetration, (iv) odourless to humans and unbearable to arthropods [139]. Generally, repellents are lipophilic volatile molecules, thus they need a suitable vehicle or formulation to be administered. 

Now only five/six compounds have been recognised and approved by the Environment Protection Agency (EPA) and the Center for Disease Control and Prevention (CDC) as active repellent ingredients. They have been admitted for skin products thanks to their low toxicity [138]. Three of them are synthetic compounds. The most known and used, since 1957, is *N*,*N*-diethyl-3-methylbenzamide (DEET). Despite its high efficacy and long-lasting effect, several studies proved its toxicity due to high skin absorption [141,142]. Its overuse may cause encephalopathy, dermal toxicity, cardiovascular diseases and psychosis and hence, its use has been now restricted and forbidden for pregnant women and children [143]. Other recognised synthetic compounds are ethyl butylacetylaminopropionate (IR3535) and picaridin. The first one is not harmful if ingested, inhaled, or used onto the skin and thus, it can be accepted for human use. Picaridin can be compared to DEET in terms of efficacy and long-lasting effect but it showed only slow toxicity [138,139].

Given the toxicity and resistance issues related to synthetic repellents, one of the biggest challenges for the scientific community is the identification of new efficient and safe compounds [142]. Since ancient times human being has used plants as means to protect himself from insects and pests, by burning or bruising them or by applying their extracts directly on the skin [144,145]. In fact, plants can produce some by-products properly to defend themselves against bloodsucking arthropods. Generally they act binding the odorant-binding proteins in the arthropod’s antennae for cuing, preventing their approach [145].

At present, research is focused on the exploitation of EOs to find out new effective natural repellents [146]. Their activity seems to be related to the presence of isoprenoid molecules. In particular, the combination of monoterpenes and sesquiterpenes in the mixture of EOs is considered to be responsible for their repellent activity [147]. Several studies reported that monoterpenes as citronellol, limonene, camphor and thymol showed effective repellent activity [148,149,150]. Citronellal and eucalyptus EOs have been recognised as skin treatments by EPA while PMD (*p*-menthane-3,8-diol), a compound of *Corymbia citriodora* (Hook.) K.D.Hill & L.A.S.Johnson. EOs, is the only natural repellent recommended by CDC, showing no adverse effects on human health [146]. Although EOs efficacy and safety have been widely proved, their use is still restricted due to some drawbacks related to their physicochemical properties. In fact, they showed rapid evaporation and a short action. Moreover, the application of pure EOs on the human skin could cause irritation [139].

To overcome these limitations the best strategy could be the encapsulation of such active ingredients to develop suitable formulations able to protect and control the release of EOs. The main systems developed for the formulation of repellent EOs are micro-/nanocapsules, MEs/NEs, liposomes, solid lipid nanoparticles and polymeric micelles [139]. Containing oily and water insoluble substances, MEs and NEs could be considered among the best choices as EOs vehicle. 

Nowadays the classical repellent formulations on the market are spray solutions and lotions. The first ones require a high amount of alcohol to solubilise the active ingredients while the second ones are emulsions with low stability. On the contrary, NEs and MEs are able to overcome these issues. In fact, they are highly stable, low viscous to be easily spread on the skin and physiologically acceptable in terms of composition [139].

Nuchuchua et al. carried out a study on NEs based on citronella (*Cymbopogon citratus* (DC.) Stapf), hairy basil (*Ocimum americanum* L.) and vetiver (*Vetiveria zizanioides* (L.) Nash) EOs [151]. They evaluated their physicochemical properties, the *in vitro* release, the *in vivo* efficacy on *Ae. aegypti* and the toxicity against normal human foreskin fibroblast (NHF) cells. They compared the different formulations before and after high-pressure homogenisation. After this high-energy process, smaller oily droplets, in the range of 150 to 160 nm, were obtained. They resulted to have a better stability, expressed as zeta potential values, after 2 months. Moreover, the small size of the oily droplets showed to play an important role in the formulation efficacy. In fact, NEs showed a higher release rate, based on a diffusion mechanism, and longer repellent activity. Authors supposed that formulations having smaller size should be able to form a whole film on the skin to prolong the activity. The best formulation was the NE composed of 10% citronella, 5% hairy basil and 5% vetiver EOs, in terms of size, stability and efficacy (4.7 h of protection). Also, Sakuluku et al. investigated the effects of high pressure homogenisation, concentration of surfactant and presence of glycerol on the physicochemical properties and mosquito repellent activity of 20% citronella EO NEs [152]. The best conditions to obtain effective NEs were as follows: concentration of surfactant at 2.5% and water:glycerol at 0:100 ratio. In fact, they demonstrated to influence the kinetic release and the activity against *Ae. aegypti*, as well as the droplet size and the long-term stability. The high amount of glycerol, and thus the high viscosity of the system, delayed the release of EOs, resulting in a prolonged repellent activity on time. 

Drapeau et al. formulated PMD based-MEs to evaluate against *Ae. aegypti* [138]. They compared a “surfactantless” ME, composed of water, propanol and PMD and a classical ME, obtained through the construction of a ternary phase diagram. The presence of surfactants led to a prolonged activity, that increased from 315 min of the “surfactantless” ME to 385 min of the classical ME, as well as the reduction of the amount of propanol. The selected formulation was composed of: 46% of H_2_O, 20% (*w*/*w*) of PMD, 25% of PrOH, 2% of Cremophor RH40 (surfactant), 3% of Texapon N70 (surfactant), 1% of 2-ethylhexane-1,3-diol (cosurfactant) and 3% of ethyl (−)-(*S*)-lactate (cosolvent). The addition of these two additives seemed to increase the activity of PMD. The cosurfactant has been selected for its repellent properties, while ethyl (−)-(*S*)-lactate could act as lactic acid competitor on human skin, a good attractant for mosquitos [153,154,155]. 

Lastly, Navayan et al. showed how MEs could be a suitable tool to prolong the repellent activity of EOs [156]. In fact, 5%, 10% and 15% eucalyptus EO-based MEs showed a protection time against Culicidae of 82, 135 and 170 min, respectively, while free EO at the same concentrations showed lower time of activity, i.e., 34, 47 and 59 min, respectively. The results obtained through the encapsulation of EO were similar to those of DEET at the same concentrations. Notably, this work outlined how nanosystems could be a desirable tool to increase EOs protection, reduce their volatility, promote their release and prolong the activity on time. 

## 5. Green Micro- and Nanoemulsions as Acaricides 

Mite control is economically important for assuring the survival of several vegetables and ornamental plants in greenhouses. For this purpose, conventional pesticides have been widely applied. They include organotin compounds, mitochondrial electron transport inhibitor-acaricides (fenazaquin, fenpyroximate, pyridaben and tebufenpyrad) and pyrethroids. Although they resulted to be very effective, their use has been limited due to the development of pest resistance and the non-target, environmental and human toxicity. These issues have highlighted the need to find out new alternatives for pest management. Botanical pesticides seem to be a valid alternative to the synthetic ones, and are in the field of acaricides products as well. In particular, EOs showed to be the most important natural sources of compounds with acaricidal activity [157,158,159,160].

Choi et al. tested the activity of fifty-three EOs against eggs and adults of *Tetranychus urticae* Koch as well as adults of the biocontrol agent *Phytoseiulus persimilis* Athias-Henriot [161]. This study revealed that the most active EOs were: caraway (*Carum carvi* L.) seed, citronella java (*Cymbopogon winterianus* Jowitt), lemon eucalyptus (*C. citriodora*), pennyroyal (*Mentha pulegium* L.), and peppermint (*M. x piperita* L.) EOs showed >90% of toxicity against adults of both mite species. From the obtained results, authors supposed that EOs were delivered and acted on the vapour phase, affecting the respiratory system of mites. 

Although their safety and effectiveness, EOs showed a short lasting effect related to their rapid volatilisation and/or degradation [125]. Thus, their encapsulation in liquid sprayable MEs and NEs could be a suitable solution. 

Concerning mite species of public health importance, Xu et al. investigated the acaricidal activity of neem oil against *Sarcoptes scabiei* expressed as the speed of kill (min) [162]. Authors compared the effectiveness of pure EO, the EO-based emulsion and the EO-based ME. Neem EO-ME demonstrated the highest acaricidal activity with a lethal time of 192 min followed by 212 min of EO-emulsion and 337 min of pure EO. As expected, the encapsulation process and the small size of the dispersed phase enhanced the activity of EOs and the interaction with target organisms. Moreover, the study reported that ME without active ingredient showed the ability to kill mites. It has been supposed that it could be due to the presence of sodium dodecyl benzene sulfonate (SDBS) in the mixture of surfactants. In fact, given its activity, it has been used to enhance the efficacy of the active ingredients [162].

Research aimed to the effective management of tick species has also been carried out. Chaisri et al. tested the activity of citronella EO on *Rhipicephalus microplus* (Canestrini) [163]. In this study, results have been expressed as larval and adult mortality. ME showed higher acaricidal efficacy compared with the pure citronella EO. In particular, larval mortality after 24 h occurred at the concentration of 0.78% EO-based ME in respect to the concentration of 3.125% of free EO. Also in this case, it could be supposed that the small size of oily droplets, <50 nm, and the presence of surfactants, Tween 20/propylene glycol 3:1, gave a synergistic effect. In particular, surfactants could interfere with the lipids of mites epicuticle, favouring the penetration of active ingredients [153,164].

dos Santos et al. proved the use of cinnamon (*Cinnamomum verum* J. Presl) EO as efficient tool to control ticks on cattle [165]. Indeed, this EO was evaluated against *R. microplus* through both *in vitro* and *in vivo* tests, the latter performed on infested dairy cows. Authors also formulated nanocapsules and NEs. They resulted to be very useful for the exploitation of cinnamon EO acaricidal activity. In fact, nanoencapsulated EO showed to be effective at low concentration (0.5%), ten times lower than that of pure EO (5%). Thus, such nanosystems at 0.5% were able to reduce infestation, oviposition and fertility of *R. microplus.* In fact, the encapsulation of EOs produced an improvement of the active ingredient stability and of its protection and guaranteed sustained release over time. 

Nevertheless, the advantages of nanotechnology cannot be ever observed. Galli et al. investigated the activity of *E. globulus* EO [166]. For the purpose, they used the same formulation, concentrations, target and procedures of those previously reported. In this case, pure EO showed to be effective decreasing the reproduction of ticks. On the contrary EO-based nanocapsules and NEs exerted low efficacy. However, it is possible to find an explanation of this result on the short exposure time (30 s) of the pests to nanosystems. This time should be not sufficient for the release of EO [167].

Mossa et al. recently investigated the acaricidal activity of emulsion and NE based on garlic (*Allium sativum* L.) EO on two eriophyid olive mites: *Aceria oleae* (Nalepa) and *Tegolophus hassani* (Keifer) [168]. After several stability studies, they found out a suitable and stable formulation, respect to the classical emulsion giving phase separation after two days. It was composed of 5% of garlic EOs, oil/Tween20 at 1:1.2 ratio and it was obtained through a sonication process for 35 min. Beyond the stability issue, garlic EO-based NE was demonstrated to be more effective than the respective emulsion. In fact, NE showed LC_50_ values of 298.22 and 309.634 μg/mL on *A. oleae* and *T. hassani*, respectively, over to 584.878 and 677.830 μg/mL of the emulsion. Moreover, they proved to be safe for mammal administration as they did not produce toxicity in rats.

Badawy et al. formulated four different NEs based on two EOs—*Callistemon viminalis* (Sol. ex Gaertn.) G.Don and *Origanum vulgare* L.—and two monoterpenes—R-limonene and pulegone [169]. They investigated the activity of 10% concentrated NEs on *T. urticae* in terms of contact toxicity, fumigant toxicity and on bean plants under greenhouse conditions. Although all the formulations showed high efficacy, the monoterpene-based NEs proved to be more toxic against the target organism and with a more rapid outbreak of the activity. Moreover, the fumigant toxicity was more pronounced than contact toxicity. As mentioned above, this could be explained by the fact that such compounds are delivered on vapour phase and act mainly on the respiratory system [161].

## 6. Green Micro- and Nanoemulsions for Developing Antiparasitic Drugs 

Micro- and nanoemulsions can also be useful tools to boost the bioactivity and increase the stability of antiparasitic drugs [170]. In the following paragraphs, we will outline the major achievements in the development of green micro- and nanoemulsions targeting both protozoan and helminth parasites.

### 6.1. Parasitic Protozoa

#### 6.1.1. *Toxoplasma gondii*

The apicomplexan *Toxoplasma gondii* (Nicolle & Manceaux) infects approximately two billion people worldwide [171]; however, seroprevalence is declining in Western Countries [172]. 

New drugs are needed for the treatment of toxoplasmosis, particularly in immunocompromised patients or in congenitally infected subjects [173]. Among the new possible drugs, atovaquone is under evaluation for its ability to suppress protozoan parasites with a broad-spectrum activity. However, the use of this drug is limited by its extremely low water solubility and bioavailability. NEs prepared with atovaquone, based on grape seed oil using spontaneous emulsification method, showed increased bioavailability and efficacy for treatment of toxoplasmosis. In fact, *in vitro* this NE resulted active against *T. gondii*, using both RH and another strain (namely, the so-called Tehran strains), cultured on HeLa cells. Such results were confirmed in *in vivo* studies in mice treated orally; these resulted with a lower number of tissue cysts compared to animals treated with the standard preparation, by virtue of better bioavailability [174].

#### 6.1.2. *Leishmania* spp.

They are vector-borne parasites belonging to *Leishmania* genus, order Trypanosomatida. They cause diseases with different clinical pictures: cutaneous (CL), mucocutaneous (MCL) and visceral (VL) [175].

Studies have been carried out on the effects of aromatic/heterocyclic sulphonamides, in the low nanomolar range, on the β-carbonic anhydrase (CA, EC 4.2.1.1) of *Leishmania* spp., which resulted effectively inhibited, without, however, any effect on parasite viability. The same drugs, formulated as NEs in clove oil, inhibited the growth of either *Leishmania infantum* Nicolle or *Leishmania amazonensis* Lainson & Shaw, being less cytotoxic than the widely used antifungal amphotericin B, as revealed by haemolytic assay [176].

NEs as a delivery system for copaiba (*Copaifera* sp. Linnaeu) and andiroba (*Carapa guianensis* Aublet) oils (nanocopa and nanoandiroba with an average particle size of 76.1 and 88.1 nm, respectively) were tested on *L. infantum* (VL) and *L. amazonensis* (CL). Nanocopa and nanoandiroba resulted toxic to promastigotes of both *Leishmania* species. In particular, ultrastructural analyses by scanning electron microscopy showed a shift of the parasite to oval shape and the retraction of flagella, as early as 1 h after treatment, with concentrations near the IC_50_ values. Furthermore, the treatment with such NEs reduced infectivity of the two species in macrophage cultures. Beneficial results were obtained also in mice experimentally infected with *L. amazonensis* or *L. infantum* (i.e., reduction in lesion size, parasite burden and inflammation). Animals affected by CL treated for eight weeks with NEs showed delay in lesion development. In VL model, around 50% reduction in parasite burden in liver and spleen of mice treated with nanocopa and nanoandiroba was found as compared with control untreated animals [177].

Nanotechnology has allowed the advancement of photodynamic therapy (PDT). In fact, many photosensitisers (PS), insoluble in water, need a nanocarrier as a physiologically acceptable carrier. NEs are efficient in solubilising liposoluble drugs, like the PS, in water. A zinc phthalocyanine (PS) oil-in-water NE, essential clove oil and polymeric surfactant (Pluronic^®^ F127) for the formulation of a topical delivery system for use in PDT was used against *L. amazonensis* and *L. infantum*. The toxicity in the dark and the photobiological activity of the formulations were evaluated *in vitro* on *Leishmania* and macrophages. The zinc phthalocyanine NE was effective in PDT against *Leishmania* spp. with several advantages compared to other topical treatments like paromomycin and amphotericin B. These drugs have many disadvantages like local side effects and a very high cost, often limiting their use [178].

The antiparasitic activity of nanoemulsionated EO of a *Lavanudula* species was tested against *Leishmania major*, a species responsible for CL. In particular, NE with EO of *L. angustifolia* Mill. (where 1,8-cineol and linalool were the major components), as well as of *Rosmarinus officinalis* L., induced significant mortality of the parasite [179]. The NE of *L. angustifolia* and *R. officinalis* EOs showed antiparasitic effects that were much more significant than those obtained with the nonemulsioned EO of *R. officinalis* [180].

A taxonomically related parasite to *Leishmania* is *Trypanosoma evansi* Steel, the etiological agent of the disease known as “Surra” and “Mal das Cadeiras” which affects horses in Brazil, and sometimes also humans. The *in vitro* trypanocidal activity of the nanoemulsified *Schinus molle* L. EO was tested; this NE reduced the number of living parasites even totally, when the highest concentration was used (1%) contrary to the non-emulsified EO, which gave only 68% of mortality as a maximum [181].

#### 6.1.3. *Plasmodium* spp.

Plasmodium parasites cause malaria, a disease which represents one of the major public health problem at global level with 219 million cases of malaria and 435,000 deaths estimated in 2017, particularly concentrated in Africa [182]. 

NEs loaded with arteether (ART), a semisynthetic derivative of artemisinin, by virtue of their solubility and consequently bioavailability, enhanced efficacy against *Plasmodium yoelii nigeriensis*, in a mouse model of experimental malaria. The *in vitro* release profile of the ART-NEs showed 62% drug release within 12 h; no significant effect on cell viability was observed. The authors focused the attention on a particular NE, loaded with ART (ART-NE), ART-NE-V, which showed a significantly enhanced bioavailability. This NE was well tolerated in the experimentally infected mice with no abnormality in behaviour, food/water consumption and general activity of the animals throughout the treatment and post treatment period. ART-NE-V, administered orally, had an 80% curative rate in comparison to the 100% cure rate achieved by intramuscular route at the same dose and to the 30% curative rate obtained in mice treated with ART in ground nut oil [183].

### 6.2. Helminths

#### *Echinococcus granulosus* 

This parasite is the aetiological agent of cystic echinococcosis (CE), a zoonotic infection with economic and public health importance worldwide distributed. CE can result in a substantial human disease burden and have a relevant economic impact on animal productivity [184,185].

EOs from *Zataria multiflora* Boiss. were tested on the cestode *Echinococcus granulosus sensu lato* [186]. The effect was tested on the protoscoleces, isolated in liver hydatid cysts collected from naturally infected sheep. NEs at different concentrations (1–2 mg/mL) induced mortality levels up to 100% after 20 and 10 min, respectively, a scolicidal activity significantly higher than that obtained with nonemulsified oil [187]. *In vivo* studies in infected mice showed that the largest cysts were significantly reduced in size, as well as their total number, in animals treated with NE, compared to those treated with nonemulsified oil [186].

The *in vitro* and *ex vivo* activity of *Melaleuca alternifolia* (Maiden & Betche) Cheel oil (tea tree oil (TTO)), its NE formulation (NE-TTO) and its major component (terpinen-4-ol) were evaluated for their effects against *Echinococcus ortleppi* (another *Echinococcus* species, also known as G5 and clearly closely related to the genotypes of *E. canadensis*). This *Echinococcus* species infects cattle, which represents the principal intermediate host, mainly distributed in Europe, Africa, some areas of Asia and South America [188]. In *ex-vivo* studies the TTO, NE-TTO and the terpinen-4-ol were directly injected in the cysts isolated from cattle. The protoscolicidal action of the TTO major compound, terpinen-4-ol, resulted very promising. In fact, just after 5 min of exposure, non-viable *E. ortleppi* protoscoleces were obtained, at the concentration of 2 mg/mL. The results obtained in this study showed protoscolicidal effect at all tested formulations and concentrations. However, the effects of TTO were higher than those of NE-TTO but this latter had the ability to reduce the volatilisation of the compound and consequently to increase the protoscolicidal effect at the action site [189].

## 7. Green Formulations against Nematodes Attacking Plants

### Meloidogyne spp.

The root-knot nematodes (*Meloidogyne* spp.) are key pests threating several crops of economic importance. Their control is mainly based on the use of chemical nematicides. However, following the withdrawal of several synthetic nematicides because of their detrimental effects on soil biodiversity, natural products of botanical origin have been investigated for their possible use against these agricultural pests. Indeed, besides effectiveness for nematode control, botanicals assure beneficial effects on structure and residual life (e.g., microorganisms) of the soil. Among the most promising natural substances with nematicidal activity, glucosinolates, isothiocyanates, aliphatic acids (e.g., acetic, butyric, hexanoic and decanoic acids), alkaloids, piperamides, flavonoids (e.g., quercetin-7-glucoside), limonoids (azadirachtin, meliacins), quassinoids (e.g., chaparrinone, glaucarubolone, klaineanone, samaderines B and E), saponins and triterpene acids (e.g., 11-oxo triterpenic, pomolic, lantanolic, lantoic, camarin, lantacin, camarinin and ursolic acids), cyanogenic glycosides, polyacetylenes, phenolic acids (e.g., salicylic, gallic, *p*-hydroxybenzoic, vanillic, caffeic, and ferulic acids), fatty acids (e.g., linoleic and oleic acids) and volatile compounds (e.g., ascaridole, 2-undecanone, furfural, benzaldehyde, thymol, geraniol, eugenol, linalool, decenal and decadienal) are the most important ones [190,191,192,193]. Among them, isothiocyanates and neem azadirachtin have been encapsulated in marketed formulations effective against the growth and development of *Meloidogyne* spp. with limited effects on soil biodiversity [193,194]. Also, the EOs from *Foeniculum vulgare* Mill., *Pimpinella anisum* L., *Eucalyptus melliodora* A Cunn ex Schauer, *Origanum vulgare* L., *O. dictamnus* L., *Mentha pulegium* L. and *Melissa officinalis* L. were effective against *M. incognita* (Kof. & White) Chitwood showing EC_50_ values of 0.2, 0.3, 0.8, 1.6, 1.7, 3.2 and 6.2 μL·mL^−1^, respectively [194,195]. Among their main constituents, benzaldehyde, γ–eudesmol, methyl chavicol, carvone, pulegone and (*E*)-anethole were ideal candidate ingredients for nematicidal formulations [194,195]. On the other hand, efforts about formulating these botanical active ingredients in micro- and nanoemulsions remain limited, outlining the urgent need of future research.

## 8. Green Micro- and Nanoemulsions in the Real World 

**Figure 3:**
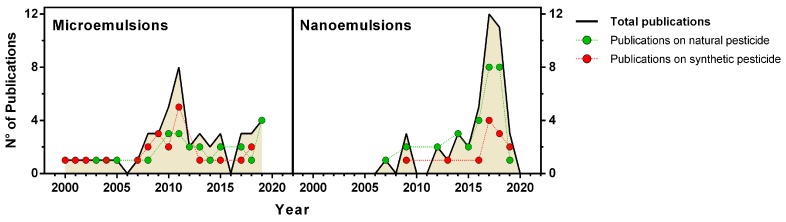
Number of publications on micro-(left) and nanoemulsion (right) vehicles for natural and synthetic pesticides per year.

As reported above, researchers in entomology and parasitology are making great efforts for the improvement of pest control in terms of efficacy and safety for environment and human being. The potential of EOs and plant extracts as biopesticides and their exploitation through nanoencapsulation opened new challenging strategies for Integrated Pest/Vector Management (IPM/IVM). From the literature analysis (Scopus database, 27 June 2019), it can be observed that in the last 20 years approximately 100 documents were published concerning the employment of MEs and NEs for the vehiculation of pesticides (Figure 3). Interestingly, MEs were firstly studied and the maximum interest was reached around 2010. On the contrary, the use of NEs as pesticide formulations was more recent, reaching the highest attention in the last 2–3 years. Another aspect to be highlighted is represented by the nature of active ingredients employed as pesticide. Regarding MEs, the use of botanical and synthetic pesticides is almost the same along the years, while for NEs there is always a stronger prevalence (~70%) of studies on natural pesticides. These results seem to highlight a temporal correlation between the diffusion of biopesticides and the development of NEs for their application. 

Even though literature reported several studies with effective results, in the real world the exploitation of EO-based MEs and NEs is still limited. Currently, the pronounced effectiveness of chemical pesticides is still predominant respect to the eco-friendly advantages of the botanical ones. However, the common awareness about the need of a more sustainable world will likely lead towards a radical change in favour of the exploitation of green solutions in the near future.

Although some patents reported the nanoformulation of chemical pesticides or the nanoencapsulation of EOs [196,197], only few of them describe EO-based MEs or NEs as biopesticides. Enan et al. patented MEs as tool for the encapsulation and delivery of two or more EOs for pest control [198]. In particular, they used unsaturated C12-C26 fatty acids and/or salts and saturated C6-C14 fatty acids and/or salts as surfactants to enhance the activity of the ingredients, resulting in an improvement of the pesticide efficacy. According to the authors, this approach brings to a reduction of the active ingredient amount required to obtain an effective pest control. 

Since scientific studies showed promising results, in the last years some botanical pesticides started to be available on the market. For example, Prev-Am^®^ Plus is a fungicide and insecticide, based on orange (*Citrus x aurantium* L.) EO, that acts for direct contact. Since Prev-Am^®^ Plus biodegrades rapidly and does not have a high environmental persistence, it is an excellent product for the Integrated Pest Management (IPM) programs, helping in the management of resistance and ensuring a minimal impact on beneficial insects. It can be used on a wide range of crops such as olive trees, vines and citrus fruits and it is allowed in organic agriculture.

Given the well-known repellent activity of EOs, a personal repellent based on EOs has been commercialised. Repel^®^ is a spray containing 30% of lemon eucalyptus EO. It was proven to be able to repel mosquitoes, in particular the vectors of Zika, West Nile, Dengue and Chikungunya viruses, for up to six hours.

Also, Bayer^®^ launched on the market Requiem^®^ EC, an emulsifiable concentrated formulation based on terpenes originally discovered in an insecticidal plant—*Dysphamia ambrosioides* (L.) Mosyakin & Clemants. It is a contact insecticide/acaricide for use in the control or suppression of many foliar-feeding species, including aphids, thrips, plant-feeding mites, whiteflies, mealy bugs, leafminers, Lygus bugs, leafhoppers and moths attacking crops such as citrus, grapes, potatoes and others. Its low toxicity on mammalian and non-target organisms makes it a reduced-risk insecticide.

## 9. Regulatory Remarks

The EU regulates the botanical products used for the control of parasites, arthropod pests and vectors through two different regulations, the EC No. 1107/2009 and the EU No. 528/2012. The first one regards the plant protection products, addressing their risk evaluation and regulating the authorisation of commercialisation in the crop protection field. The second one, named Biocidal Products Regulation (BPR), takes into account “any substance or mixture exerting a controlling effect on any harmful organism by any means other than mere physical or mechanical action”. 

Interestingly, while EC No. 1107/2009 does not mention nanomaterials at all, the BPR poses specific issue, stating that “where nanomaterials are used in that product, the risk to human health, animal health and the environment has been assessed separately”. For this reason, BPR excludes the possibility of “simplified authorisation procedure” followed for “low-risk” products, in the case of biocide containing nanomaterials. Moreover, the BPR highlights the necessity of a proper methodology for the risk evaluation for nanomaterials.

Although nanotechnology showed to be a great opportunity to achieve a more rational Integrated Pest Management (IPM), the lack of knowledge on the fate and effects on humans and environment of nanomaterials represents, nowadays, an important limitation on their widespread exploitation. It is needed an increased regulatory oversight to ensure their appropriate identification and risk assessment evaluation. In this direction, the European Community is addressing innovative methodologies able to evaluate the risk of nanopesticides and nanomaterials in general. In particular, the European Chemicals Agency (ECHA) is starting to define the guidelines for the monitoring and the evaluation of nanomaterials in the environment, and for the support about their registration procedure (four appendices for nanomaterials applicable to Chapters R.6, R.7a, R.7b and R.7c of the IR&CSA guidance) [199]. 

Among the different risk assessment procedures, the Quantitative Structure-Activity Relationship/Quantitative Structure-Property Relationship (QSAR/QSPR) appears one of the most promising tools for chemicals. In this regard, the scientific community is moving towards an innovative tool, nano-QSAR/QSPR, introducing the computational approach in the risk assessment of nanomaterials. Several studies focused on how nano-QSAR/QSPR should be supported by the development of new interpretative descriptors for the nanosystems. Moreover, they highlighted the need to model different classes of nanomaterials, given their wide variability in the molecular structure and mechanism of toxicity [200,201]. 

Currently, the most studied nanomaterials through nano-QSAR/QSPR for risk evaluation are metal oxide and carbon nanoparticles [202,203,204]. 

Although nano-QSAR/QSPR is showing to be a useful approach on the risk assessment on nanomaterials, it should be improved by increasing the experimental data on the toxicity of all the different nanomaterials classes, that are still restricted, allowing nano-QSAR/QSPR to be a real tool for the prediction of nanomaterials fate. 

Even though much progress has been made, the efforts that are underway to improve the risk assessment procedures of nanomaterials should continue. A pragmatic and internationally accepted nanomaterial decision framework is necessary in order to clarify all the potential toxicological issues, opening to a large-scale diffusion of all the nano-based products.

## 10. Conclusions and Key Challenges for Future Research

Control of pests and vectors is a highly current issue since they are known to affect the health of the planet. Acting as vectors of devastating pathogens, many pests constitute a threat for the health and survival of living beings, as plants, animals and, above all, human beings. Although in the last decades chemical pesticides have been considered the solution to this problem, nowadays we are becoming aware that they are nothing more than a palliative. In fact, their efficacy has been overshadowed by two main drawbacks, the environmental hazards and the resistance development, linked to their overuse. 

Nowadays, a possible solution has been found on the exploitation of botanical compounds, in particular EOs, which showed to possess antiparasitic, insecticidal, larvicidal, acaricidal, ovicidal, fumigant, repellent and chemosterilant effects among other biological properties. They could ensure a sustainable and eco-friendly way to control parasite and pest spreading. In this direction, several efforts have been done in the scientific research fields. For example, several botanical species have been deeply investigated to find out a high number of new active compounds. Anyway, suitable and innovative solutions could be reached only through a multidisciplinary approach. In fact, the physicochemical limits of biological compounds could be overcome only thanks to the development of suitable formulations. For this reason, technological research could offer the real solution to exploit the great advantages and the effectiveness of botanical compounds. Besides insecticides and acaricides, this is also true also for the development of new nematicides, as well as to develop drugs against parasites of public health importance.

In this scenario, nanotechnologies represent the tool of choice. Since they can encapsulate the active compound in a suitable way to protect them and, at the same time, to exalt their efficacy, botanical compound-based nanosystems could represent the turning point in the pest management. Among the different nanosystems available, the MEs and NEs proved to be the most suitable as vehicles for botanicals when those are characterised by high lipophilicity. 

Although promising results have been reported in the literature, a strong gap between the theoretical research and the practical application still persists. In this direction, in the near future it is necessary to improve and examine in-depth different aspects of green nanotechnologies; in particular, (i) industrialisation of botanical species plantation in order to increase the amount and the yield of active ingredients, (ii) standardisation of products in terms of quali-quantitative composition, (iii) optimisation of the formulation process to enhance the stability and efficacy of nanosystems, (iv) reduction of the costs of production, (v) evaluation of the real long-term effects of the new products on the environment and non-target organisms and (vi) definition of a clear normative framework able to facilitate the commercial authorisation of botanical compound-based nanosystems.
